# Optimization for ultrasonic-microwave synergetic extraction of total iridoid glycosides and screening of analgesic and anti-inflammatory active fractions from *patrinia scabra* Bunge (Valerianaceae)

**DOI:** 10.1186/s12906-021-03489-7

**Published:** 2022-01-04

**Authors:** Quhuan Ma, Yanmei Lu, Yi Deng, Xiaodong Hu, Wanyu Li, Hongzhen Jia, Yuer Guo, Xiaofeng Shi

**Affiliations:** 1 School of Pharmaceutical Science, Gansu University of Traditional Chinese Medicine, Lanzhou, 730000 Gansu China; 2 Gansu Provincial Academy of Medical Sciences, Lanzhou, 730000 Gansu China; 3 Laboratory of Pharmacology, Lanzhou University, Lanzhou, 730000 Gansu China; 4 Gansu Light Industry Science Research Institute Co., Ltd, Lanzhou, 730000 Gansu China

**Keywords:** *Patrinia scabra*, Total iridoid glycosides, Ultrasonic-microwave synergetic extraction, Screening of active fractions, Analgesic, Anti-inflammatory

## Abstract

**Background:**

*Patrinia scabra* Bunge is a well-known herbal medicine for its favorable treatment on inflammatory diseases owing to its effective ingredients, in which iridoid glycoside plays an extremely significant role. This article aimed to improve the content of total iridoid glycosides in crude extract through a series optimization of extraction procedure. Moreover, considering that both pain and inflammation are two correlated responses triggered in response to injury, irritants or pathogen, the article investigated the anti-inflammatory and analgesic activities of *P. scabra* to screen out the active fraction.

**Method:**

*P. scabra* was extracted by ultrasonic-microwave synergistic extraction (UMSE) to obtain total iridoid glycosides (PSI), during which a series of conditions were investigated based on single-factor experiments. The extraction process was further optimized by a reliable statistical method of response surface methodology (RSM). The elution fractions of *P. scabra* extract were prepared by macroporous resin column chromatography. Through the various animal experiment including acetic acid-induced writhing test, formalin induced licking and flinching, carrageenan-induced mice paw oedema test and xylene-induced ear edema in mice, the active fractions with favorable analgesic and anti-inflammatory effect were reasonably screen out.

**Results:**

The content of PSI could reach up to 81.42 ± 0.31 mg/g under the optimum conditions as follows: ethanol concentration of 52%, material-to-liquid ratio of 1:18 g/mL, microwave power at 610 W and extraction time of 45 min. After gradient elution by the macroporous resin, the content of PSI increased significantly. Compared with other concentrations of elution liquid, the content of PSI in 30 and 50% ethanol eluate was increased to reach 497.65 and 506.90 mg/g, respectively. Owing to the pharmacology experiment, it was reasonably revealed that 30 and 50% ethanol elution fractions of *P. scabra* could relieve pain centrally and peripherally, exhibiting good analgesic and anti-inflammatory activities.

**Conclusion:**

*Patrinia scabra* possessed rich iridoids and exhibited significant analgesic and anti-inflammatory activities.

## Background

*Patrinia scabra* Bunge, a perennial herb locally known as “*mu tou hui*” belonging to the Valerianaceae family. *P. scabra* is widely distributed in midwest of China and is famous for its favorable treatment on various diseases such as malaria, dysentery, leukemia, gastric cancer, typhoid fever, injuries from falls, cervical erosion [1, 2]. Modern research has found that its main chemical constituents involve volatile oil, iridoids and its glycosides, phenylpropanoids, lignans, sesquiterpenes, triterpenes, steroids, coumarins and flavonoids [2, 3]. In the last few decades, 55 iridoids and iridoid glycosides have been identified in the genus patrinia [4]. With the development of separation analysis technology, more and more iridoid monomeric compounds have been successfully isolated from *P. scabra* [5–9].

The published work of “Essentials of Chinese Materia Medica and Medical Formulas” revealed that *P. scabra* has a good therapeutic effect on various inflammatory diseases including leucorrhea, diarrhea, malaria, jaundice, intestinal abscesses and ulcers [10]. The study on anti-inflammatory activity of *P. scabra* has attracted great attention from researchers. There was reported that 70% ethanol extraction of the roots from *P. scabra* exhibited anti-inflammatory activity towards lipopolysaccharide (LPS)-induced nitric oxide (NO). Two novel iridoids and one reported patrineolignan B have been found to significantly decrease the LPS-induced NO production in a concentration-dependent manner [11]. Considering the chemical structure of iridoid, it was speculated that the ethyl group in the iridoids may play a role in the anti-inflammatory effects [2]. In addition, both of patrineolignan B and patriscabrin F (a novel chlorinated iridoid) in *P. scabra* were found to play an anti-inflammatory role under the same molecular mechanism of action [12, 13]. It could be clearly seen from the above that the iridoid in *P. scabra* has good research value and application prospect in anti-inflammatory. Moreover, in the process of in-depth study on inflammation mechanism, we surprisingly found some interesting researches about *P. scabra* on analgesia. The functional indications of Chinese Materia Medica Dictionary has recorded that the *mu tou hui* could cure the pain of bone joints in limbs [14]. *Patrinia villosa*, a herbal medicion belonging to the same category of *P. scabra*, was reported to possess strong analgesia effect [15]. The action mechanisms of pain and inflammation are closely related, both of which are two immune responses triggered in response to injury, irritants or pathogen [16]. Inappropriate inflammation can result in physical pain and pain always accompanies with the occurrence of inflammatory [17, 18]. However, a few literatures focused on the analgesic activity of *P. scabra* in vivo which should arise people’s attention. Besides, the iridoid in *P. scabra* was preliminary considered to be crucial role not only in inflammatory but also in analgesic activity. Thus, multiple component analysis steps for herbal medicine including optimization of extraction techniques, confirmation of component analysis and validation of statistical methods should be combined to comprehensively investigate the properties of iridoid in *P. scabra.*

In present study, *P. scabra* was extracted by Ultrasonic-Microwave synergistic extraction. Based on single-factor and response surface methodology, a series of reaction conditions were optimized to improve the content of total iridoid glycosides in *P. scabra* extract. The macroporous resin column chromatography was applied to further enrich the content of total iridoid glycosides. The total content of iridoid glycosides in different elution fractions of *P. scabra* extract were compared and it was found that 30 and 50% ethanol eluate showed good elution effect. Various animal experiments including acetic acid-induced writhing test, formalin induced licking and flinching, carrageenan-induced mice paw oedema test and xylene-induced ear edema in mice were established to investigate the analgesic and anti-inflammatory effect of elution fractions. It was reasonably confirmed that the iridoid glycosides-rich elution fraction had favorable analgesic and anti-inflammatory effect.

## Methods

### Chemicals and reagents

Morroniside standards (20 mg, Shanghai Shifeng Biotechnology Co., Ltd., batch number: 20140419, purity ≥98%); Aspirin enteric-coated tablets (Shiyao group Ouyi Pharmaceutical Co., Ltd); Ethanol absolute (Tianjin Beilian Fine Chemicals Development Co., Ltd); Acetic acid (Tianjin Taixing Reagent Factory); 37–40% formaldehyde solution (Tianjin Best Chemical Co., Ltd.); Carrageenan (Shanghai McLean biochemical Technology Co., Ltd.); Xylene (Tianjin Baishi Chemical Co., Ltd.).

### Plant material

*P. scabra* was collected in May 2019 from Lanzhou suburb (Gansu, China) and identified by Professor Chang-Shuan Shi who majored in plant classification (Gansu Provincial Academy of Medical Sciences, Lanzhou, China). A voucher specimen (GSAMCVS-2019.0802) was deposited in plant herbarium of Gansu Provincial Academy of Medical Sciences [8].

### Experimental animals

Male adult Kunming mice (20 ~ 30 g) were obtained from Medical Experimental Animal Center of Lanzhou University (approval number: SCXK (GAN), 2018–0002). All experimental protocols with animals in this study were approved by the Experimental Animal Ethics Committee (EAEC) of Gansu University of Traditional Chinese Medicine (Reference No. 2020–296). Animals were randomly housed with free access to food and water and were kept in rooms with temperature maintained at 25 °C and humidity at 60–70% in a 12-h light/ dark cycle.

### Standard curve drawing

Based on the determination method established by our research group [19], the concentration (X, mg/mL) was plotted against the absorbance (Y). The linear regression equation was obtained as follows: Y = 28.44805X + 0.00004, r^2^ = 0.9994. Results indicated that there was a good linear relationship between the absorbance and concentration when the morroniside concentration ranged from 0.0055 to 0.033 mg/mL.

### Determination of total iridoid glycosides content

*P. scabra* extraction (1 mL) was precisely transferred and the absorbance was determined. The concentration was calculated by the above linear regression equation, and then the content of total iridoid glycosides was calculated according to the following formula:

Total iridoid glycosides content (mg/g) = total iridoid glycosides content in the test solution (mg) / weight of *P. scabra* powder (g).

### Ultrasonic-microwave synergistic extraction process of PSI

*P. scabra* was dried at 65 °C for 12 h in the electric thermostat oven, and was powdered by a high-speed pulverizer. The power was passed through sieve of 272 μm aperture (40 mesh). The *P. scabra* powder (2 g) was accurately weighed and placed in 250 mL corked conical flask. Then different concentrations of ethanol solution were added under the ratio of material to liquid, and extracted for a designed time at 60 °C by ultrasonic-microwave synergistic extraction device. The extract was filtered and quantitatively adjusted to 100 mL with the same concentration of ethanol solvent. Thereafter, 1 mL of the extraction was taken for the determination of total iridoid glycosides.

### Single factor experimental design

The extraction conditions were studied with the ethanol concentration of 30–70%, material-to-liquid ratio of 1:14–1:22 g/mL, microwave power of 300–700 W and extraction time of 30–55 min [20].

### Experiment design of RSM

A Box-Behnken experimental design with four factors and three levels was established on the basis of single factor experiment by the principle of central composite design. The ethanol concentration (A), material-to-liquid ratio (B), microwave power (C) and extraction time (D) were taken as response variables, and the content of total iridoid glycosides was taken as response value. The mathematical model of fitting Quadratic Polynomials by least Square algorithm was established before the optimal process parameters of PSI were obtained by this model. Twenty-nine combinations were designed by the RSM optimization test as shown in Table 1.

**Table 1:** Factors and Levels of Box-Behnken

Levels	Factor			
	A	B	C	D
−1	40	1:16	500	40
0	50	1:18	600	45
1	60	1:20	700	50

Note: A ethanol concentration (%), B material-to-liquid ratio (g.mL^−1^), C: microwave power (W), D extraction time (min)

### Preparation of different elution fractions from *Patrinia scabra*

The *P. scabra* extraction prepared under optimal extraction conditions was separated by macroporous resin column chromatography with the gradient elution consisting of water, 10, 30, 50 and 70% ethanol. The extract of different elution fractions were then dried by vacuum concentration and evaporation. And then, the content of PSI from each elution fraction was measured through the equation: Y = 28.44805X + 0.00004. The suspensions of required concentration were dissolved in distilled water before animal experiments. Abbreviations were as follows: water elution fraction (EW), 10% ethanol elution fraction (E10), 30% ethanol elution fraction (E30), 50% ethanol elution fraction (E50), 70% ethanol elution fraction (E70).

### Acetic acid-induced writhing test

Acetic acid-induced writhing test was carried out according to the method described previously [21]. The 140 mice were randomly divided into 14 groups. The positive control group was treated with aspirin (500 mg/kg) and saline served as negative control. The treatment groups consisted of EW (100 mg/kg, 200 mg/kg), E10 (100 mg/kg, 200 mg/kg), E30 (50 mg/kg, 100 mg/kg, 200 mg/kg), E50 (50 mg/kg, 100 mg/kg, 200 mg/kg), E70 (100 mg/kg, 200 mg/kg). The animals in each group received intragastric administration once a day for successive 6 days with the dose of 0.2 ml/20 g. Ten minutes after the last administration, the animals were intraperitoneally injected with 0.6% glacial acetic acid and immediately put into the behavior cage for videoing via a camera under the cage attached to a computer. The abdominal writhing times of mice were observed for thirty minutes through the video. It was a complete writhing phenomenon when the mice had such behavioral reactions as abdomen concaving, trunk and hind limbs stretching, buttocks lifting.

### Formalin induced licking and flinching

The formalin-induced pain test was evaluated as previously described [22], with a slight modification. The mode of administration and animal groups were the same as the acetic acid-induced writhing test. Ten minutes after the last oral drug administration, each mouse received an intra-plantar injection of 25 μL formalin (5%, now used) in the right-hind paw and was immediately placed into the behavior cage for one-hour behaivoral videoing. Based on the previous method and made some modifications [23], the amount of time spent and frequency of licking /flinching of the injected paw was measured for each 5-min time in 1 h through the video.

### Carrageenan-induced mice paw oedema test

The paw oedema test was performed according to the method described previously [24, 25], with some modifications. The drug administration mode and animal groups were the same as the acetic acid-induced writhing test. Ten minutes after the last administration, the initial perimeter and thickness of left-hind paw were measured with a digital micrometer before subcutaneous intraplantar injection of 1% carrageenan (0.03 mL, suspended in sterile 0.9% saline) into the left hind paw. An hour after the injection of carrageenan, the perimeter and thickness of swollen paw were measured. As the whole experiment was processed in two batches by the same person in order to avoid the error due to operation, it all altogether spent about 130 min. The paw edema and thickening rates were calculated according to the following formula:

Paw edema rate (%) = (post − inflammatory perimeter − initial perimeter)/initial perimeter × 100% Paw thickening rate (%) = (post − inflammatory thickness − initial thickness)/initial thickness × 100%

### Xylene-induced ear edema in mice

Xylene-induced ear edema test was carried out on the basis of the previous method with minor modifications [26]. The drug administration mode and animal groups were the same as the acetic acid-induced writhing test. 10 min after the last oral drug administration, the topical application of 100% 50 μL of xylene to the anterior and posterior surfaces of the right ear lobe was performed, while the left ear was used as control. 40 min after xylene application, the mice were euthanized by an overdose of anesthesia. Both ears were excised immediately by using a perforator with a diameter of 8 mm, and then measured for weight and thickness with an analytical balance and digital micrometer, respectively. As the whole experiment was processed in three batches by the same person in order to avoid the error due to operation, it all altogether spent about 200 min. The ear swelling and thickening rates were calculated according to the following formula:

$$\mathrm{Ear}\ \mathrm{edema}\ \mathrm{rate}\ \left(\%\right)=\left(\mathrm{right}\ \mathrm{ear}\ \mathrm{weight}-\mathrm{left}\ \mathrm{ear}\ \mathrm{weight}\right)/\mathrm{left}\ \mathrm{ear}\ \mathrm{weight}\times 100\%.$$

$$\mathrm{Ear}\ \mathrm{thickening}\ \mathrm{rate}\ \left(\%\right)=\left(\mathrm{right}\ \mathrm{ear}\ \mathrm{thickness}-\mathrm{left}\ \mathrm{ear}\ \mathrm{thickness}\right)/\mathrm{left}\ \mathrm{ear}\ \mathrm{thickness}\times 100\%$$

### Statistical analysis

All the experimental data were analyzed using SPSS 18.0 software and expressed as mean ± standard error of mean (SEM). Kruskal-Wallis test was used for statistical evaluation of the data, and pairwise comparison between groups was performed. The values of *p* <  0.05 were considered to be statistically significant (^*^P <  0.05*,* ^**^P <  0.01, and ^***^P <  0.001).

## Results

### Single factor experiment

**Fig. 1:**
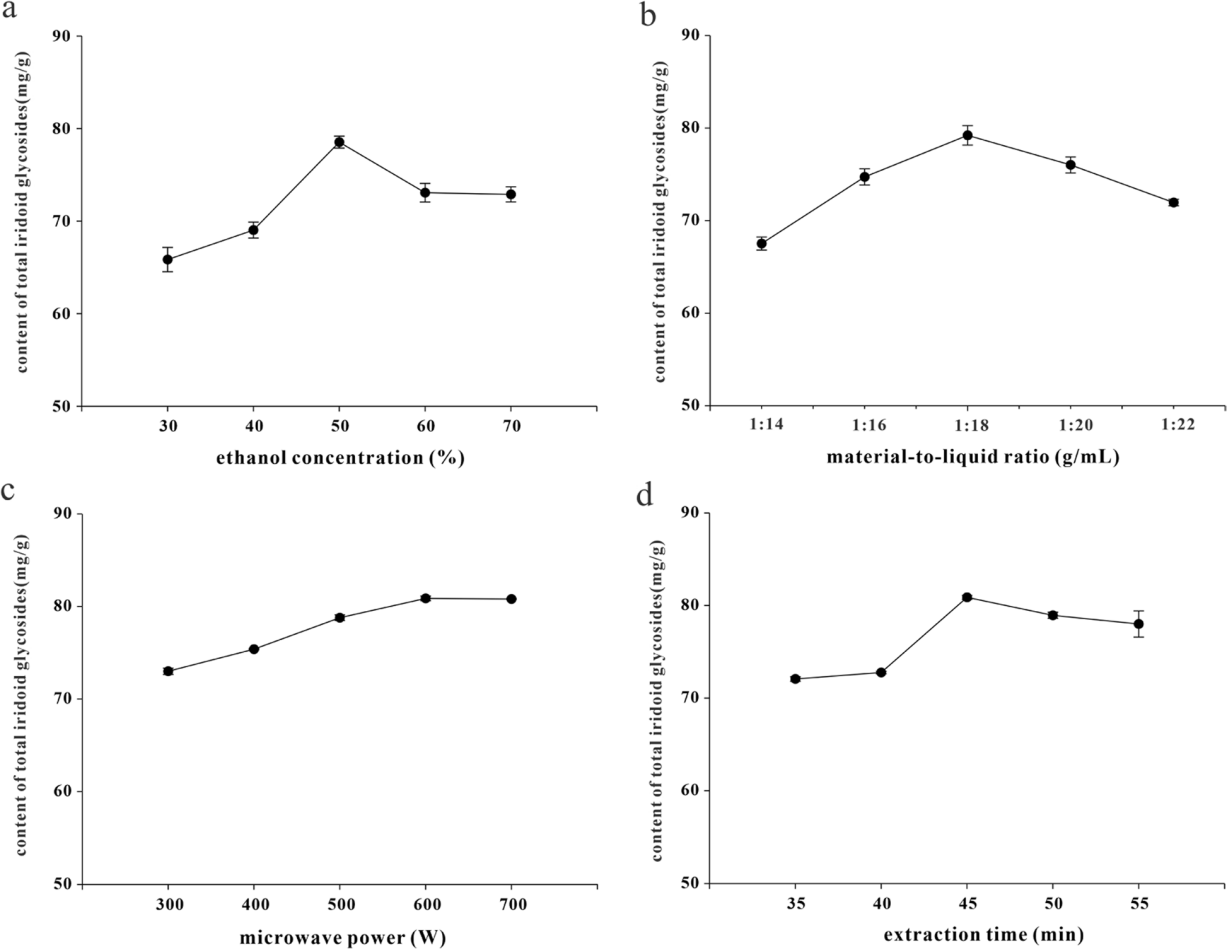
Effect of ethanol concentration (%), material-to-liquid ratio (g/mL), microwave power (W) and extraction time (min) on the extraction content of total iridoid glycosides from *patrinia scabra* (*n* = 3)

Aqueous ethanol of different concentrations was used to conduct the single factor analysis under the following conditions: material-to-liquid ratio of 1:20 g/mL, microwave power at 500 W, extraction time kept at 50 min, respectively. As shown in Fig. 1a, the content of PSI significantly increased with the ethanol concentrations increasing from 30 to 50%, indicating that a certain concentration of ethanol solvent was conducive to the dissolution of total iridoid glycosides. However, the content of total iridoid glycosides was significantly decreased when the ethanol concentration exceeded 50%. It was possibly because that other alcohol-soluble or lipid-soluble substances began to dissolve with the increase of alcohol concentration. Therefore, it was suitable to select 50% ethanol concentration.

In order to explore the effect of the material-to-liquid ratio, other influencing factors such as ethanol concentration, microwave power and extraction time were kept at 50%, 500 W and 50 min, respectively. The content of PSI was significantly increased with the increase of material-to-liquid ratio, while it was significantly decreased when the material-to-liquid ratio was further enhanced (Fig. 1b). This was due to that the material-to-liquid ratio within the appropriate scope was beneficial to the dissolution of total iridoid glycosides. However, the concentration of alcohol solution was diluted when the material-to-liquid ratio exceeded a certain value, which reduced the extraction effect of PSI. Therefore, the ratio of material to liquid was determined to be 1: 18 g/mL.

The effect of microwave power on total iridoid glycosides was investigated when the ethanol concentration, material-to-liquid ratio and extraction time were kept at 50%, 1:18 g/mL, and 50 min, respectively. As shown in Fig. 1c, the content of PSI was significantly increased when the microwave power was increased from 300 W to 600 W, and dropped slightly at higher microwave power. Possibly, the rising speed of the extraction system temperature was accelerated with the increase of microwave power, thereby contributing to the rapid dissolution of PSI. The temperature of the extraction system could have been able to make the total iridoid glycosides in *P. scabra* dissolve fully when the microwave power increasing to 600 W. Therefore, it was appropriate to choose 600 W microwave power.

Total iridoid glycosides with different extraction time were evaluated when the ethanol concentration, material-to-liquid ratio, and microwave power were kept at 50%, 1:18 g/mL, and 600 W, respectively. The content of PSI increased with the extraction time rising from 35 min to 45 min, while dropped with longer extraction time (Fig. 1d). This was because that the ultrasonic wave could produce strong vibration, high acceleration and strong cavitation effect in the material, which has a strong effect on plant cells and molecules, thus rapidly increasing the content of total iridoid glycosides. However, the structure of total iridoid glycosides would be destroyed if the ultrasonic time was too long, which might cause the decrease of extraction yield. Therefore, the appropriate extraction time was determined to be 45 min.

### Experimental design and results of box-Behnken

The four-factor optimization experiments of the process were designed by using Box-Behnken design method on the basis of single-factor experiment. The experimental design and results were shown in Table 2.

**Table 2:** Experimental Design and Results of Box-Behnken

Number	A	B	C	D	Y
1	−1	0	1	0	75.44
2	0	0	0	0	80.38
3	0	1	−1	0	74.02
4	0	−1	0	1	70.38
5	0	0	1	1	80.13
6	0	0	−1	1	79.46
7	0	−1	0	−1	70.11
8	0	−1	1	0	69.73
9	0	0	0	0	80.58
10	0	0	0	0	80.72
11	−1	0	0	1	75.98
12	0	−1	−1	0	69.05
13	1	1	0	0	72.49
14	0	0	1	−1	79.68
15	1	0	0	1	78.85
16	−1	1	0	0	70.73
17	0	0	0	0	81.07
18	1	0	−1	0	77.74
19	0	1	1	0	74.47
20	−1	0	0	− 1	76.28
21	1	0	0	−1	77.55
22	1	−1	0	0	67.77
23	1	0	1	0	77.17
24	0	0	−1	−1	79.03
25	−1	−1	0	0	65.66
26	−1	0	−1	0	75.05
27	0	0	0	0	80.87
28	0	1	0	-1	74.73
29	0	1	0	1	74.91

Note: A ethanol concentration (%), B material-to-liquid ratio (g/mL), C microwave power (W), D extraction time (min), Y content of total iridoid glycosides (mg/g)

Multiple regression fitting was performed on the test data in Table 2 by using Design Expert11.1.2 software to obtain the quadratic polynomial regression equation of PSI on ethanol concentration, solid-liquid ratio, microwave power and extraction time.

Y = 80.72 + 1.04A + 2.39B + 0.1902C + 0.1937D-0.0853AB-0.2379 AC + 0.4013 AD-0.0577 BC-0.023BD + 0.0037CD-3.46A^2^–8.04B^2^–0.9227C^2^–0.1595D^2^.

The statistical significance of the regression model was checked by *F*-test and *p*-value, and the analysis results of the model and significance test of the model coefficient are shown in Table 3.

**Table 3:** Analysis of Variance of the Regression Model

Variance source	Sum of squares	Degree of freedom	Mean square	***F*** value	***P*** value
Model	549.8	14	39.27	665.73	< 0.0001^**^
A	12.86	1	12.86	217.93	< 0.0001^**^
B	68.44	1	68.44	1160.19	< 0.0001^**^
C	0.4342	1	0.4342	7.36	0.0168^*^
D	0.4503	1	0.4503	7.63	0.0153^*^
AB	0.0291	1	0.0291	0.493	0.4941
AC	0.2264	1	0.2264	3.84	0.0703
AD	0.6442	1	0.6442	10.92	0.0052^**^
BC	0.0133	1	0.0133	0.226	0.6418
BD	0.0021	1	0.0021	0.0358	0.8526
CD	0.0001	1	0.0001	0.0009	0.9763
A^2^	77.45	1	77.45	1312.91	< 0.0001^**^
B^2^	419.07	1	419.07	7104.14	< 0.0001^**^
C^2^	5.52	1	5.52	93.61	< 0.0001^**^
D^2^	0.1649	1	0.1649	2.8	0.1167
Residual	0.8259	14	0.059		
Lack of Fit	0.5438	10	0.0544	0.7710	0.6649
Pure error	0.2821	4	0.0705		
Total deviation	550.62	28			
R^2^ = 0.9985 R_adj_^2^ = 0.9970					

Note: A ethanol concentration (%), B material-to-liquid ratio (g.mL^−1^), C microwave power (W), D extraction time (min), ** represents extremely significant (*P* < 0.01), * represents significant (*P* < 0.05)

As shown in Table 3, the quadratic regression model was very significant when the F value was 665.73 (*P* < 0.0001), while the difference of the lack of fit was not significant when the *P* value was 0.7710 (*P* > 0.05). The determination coefficient of the model (R^2^ = 0.9985) indicated that the model was reliable and 99.85% of the variability of experimental data could be explained by this regression model. The correction coefficient (Radj^2^ = 0.9970) indicated that 99.70% of the experimental results were affected by the four factors. All these results illustrated that the experimental data could be described by this regression model, and the unknown factors had little influence on the experimental results. The equation reflected the relationship between the content of PSI and ethanol concentration, ratio of material to liquid, microwave power and extraction time, and could well predict the variation of PSI with various parameters.

In conclusion, this model was adequate to analyze and predict the content of total iridoid glycosides. The effect of each argument on the dependent variable could be judged by the F value in Table 3, which was in the following order: B > A > D > C. The greatest factor that influenced the content of total iridoid glycosides in *P. scabra* was material-to-liquid ratio (*P* < 0.01), following by ethanol concentration (P < 0.01), extraction time (*P* < 0.05) and microwave power (P < 0.05). According to the regression equation and analysis of variance, the effects of A^2^、B^2^ and *C*^*2*^ on the extraction of total iridoid glycosides were extremely significant (P < 0.01), and the interaction between ethanol concentration and extraction time was significant.

### Result analysis of response surface

**Fig. 2:**
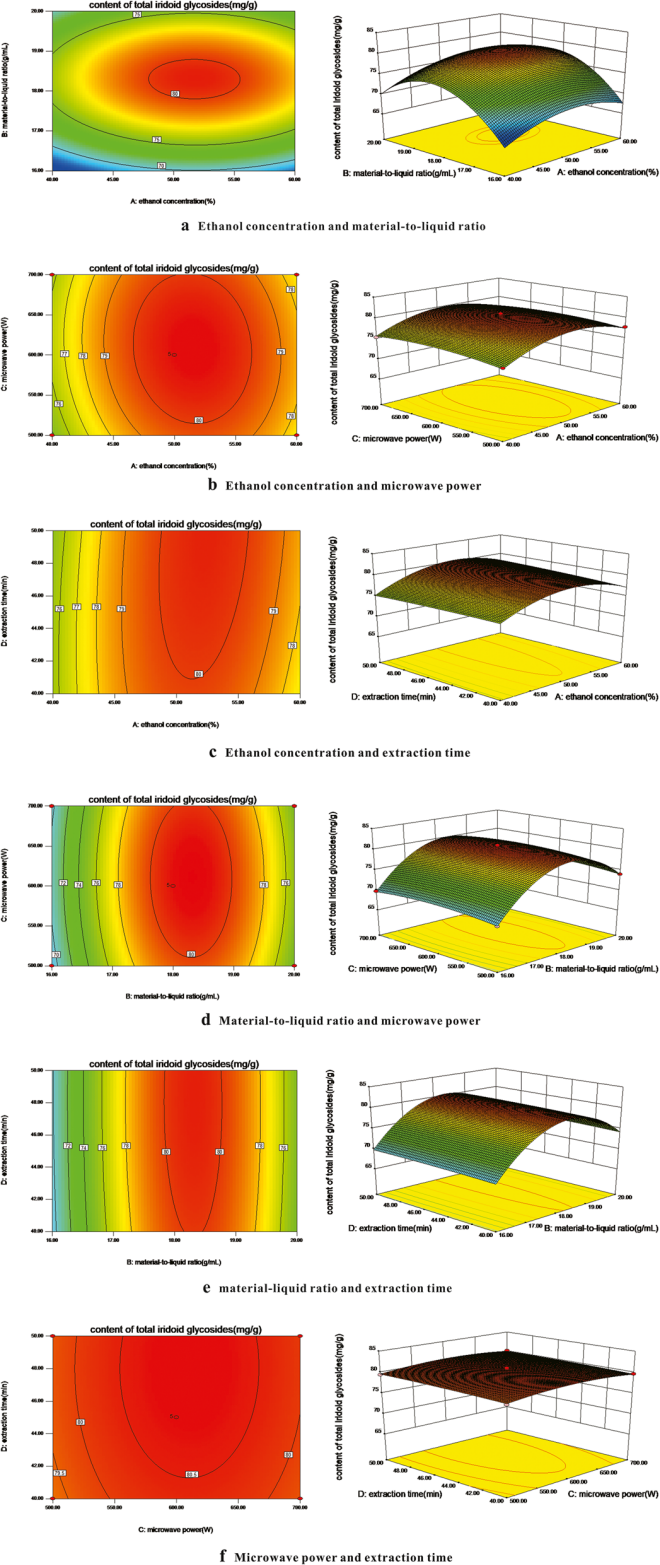
Response surface diagrams of the effect of ethanol concentration (%), material-to-liquid ratio (g/mL), microwave power (W), extraction time (min) and their interactions on the content of total iridoid glycosides from *P. scabra.* **a** Ethanol concentration and material-to-liquid ratio. **b** Ethanol concentration and microwave power. **c** Ethanol concentration and extraction time. **d** Material-to-liquid ratio and microwave power. **e** Material-liquid ratio and extraction time. **f** Microwave power and extraction time

Figure 2 showed the 3-D response surface plot and contour plot of the effects of ethanol concentration, material-to-liquid ratio, microwave power, extraction time and their interaction on the PSI content.

Figure 2a showed the effect of ethanol concentration and material-to-liquid ratio on the total iridoid glycosides content. As shown from the 3-D response surface plot and contour plot, the contour linear density moving along the material-to-liquid ratio to the peak direction was significantly higher than that along the ethanol concentration direction. The response surface curve in the direction of ethanol concentration was overall smooth, while was steeper in the direction of material-to-liquid ratio. These results indicated that the effect of material-to-liquid ratio on total iridoid glycosides content was greater than that of ethanol concentration.

Figure 2b showed the effect of ethanol concentration and microwave power on the content of total iridoid glycosides. The contour plot indicated that the contour linear density moving along the microwave power to the peak direction was obviously lower than that along the ethanol concentration direction. The 3-D response surface plot also indicated that the slope of the response surface curve in the ethanol concentration direction was larger than that in the microwave power direction, suggesting that the effect of ethanol concentration on total iridoid glycosides content was higher than that of microwave power.

Figure 2c showed the effect of ethanol concentration and extraction time on the content of total iridoid glycosides. According to the contour plot, the contour density moving along the ethanol concentration to the peak direction was higher than the extraction time direction. In the 3-D response surface plot, the steepness of the response surface curve in the extraction time direction was less than that in the ethanol concentration direction. Therefore, the ethanol concentration had a greater effect on the content of total iridoid glycosides compared with the extraction time.

Similarly, Fig. 2d, e and f showed the effects of material-to-liquid ratio and microwave power, material-to-liquid ratio and extraction time, microwave power and extraction time on the content of total iridoid glycosides, respectively. According to the contour plot and the 3-D response surface plot, their effects on the total iridoid glycosides content were as follows: the material-to-liquid ratio was greater than microwave power, the material-to-liquid ratio was larger than extraction time, and the microwave power was greater than extraction time. Response surface analysis reflects the influence of various factors directly, and its effect is better than single factor test.

### Model verification

According to the results of Box-Behnken test and combined with the regression model, the optimal process conditions for ultrasonic-microwave synergetic extraction of PSI were predicted as follows: 51.95% of ethanol concentration, 1:18.29 g/mL of material-to-liquid ratio, 607.56 W of microwave power, and 49.21 min of extraction time. Under these conditions, the content of total iridoid glycosides was 81.09 mg/g. In order to verify the predictability of the response surface model and simultaneously simplify the experimental operation, the optimum extraction conditions of PSI were optimized as follows: 52% of ethanol concentration, 1:18 g/ml of material-to-liquid ratio, 610 W of microwave power, and 50 min of extraction time. Three parallel tests were performed under these conditions, and the content of total iridoid glycosides reached 81.42 ± 0.31 mg/g with RSD of 0.38%, and the error between the predicted value of the model was 0.41%. The results indicated that the model could effectively predict the content of total iridoid glycosides in the extraction process. The optimized results were reliable, and could be used to guide the extraction technology of PSI by ultrasonic-microwave synergetic extraction.

### Content determination of PSI from different elution fractions

The contents of PSI from each elution fraction were shown in Table 4. The order of PSI content in different fractions was as follows: E50 > E30 > E70 > E10 > EW.

**Table 4:** The content of total iridoid glycosides from *Patrinia scabra* (mg/g)

Number	Elution fraction				
	EW	E10	E30	E50	E70
1	117.86	202.51	490.75	505.16	400.91
2	109.08	208.30	497.10	517.07	416.45
3	114.11	209.76	505.10	498.48	412.23
Average	113.79	206.86	497.65	506.90	409.86

Note: E10 10% ethanol elution fraction, E30 30% ethanol elution fraction, E50 50% ethanol elution fraction, E70 70% ethanol elution fraction

### Effects of different elution fractions from *P. scabra* on the acetic acid-induced writhing times of mice

**Fig. 3:**
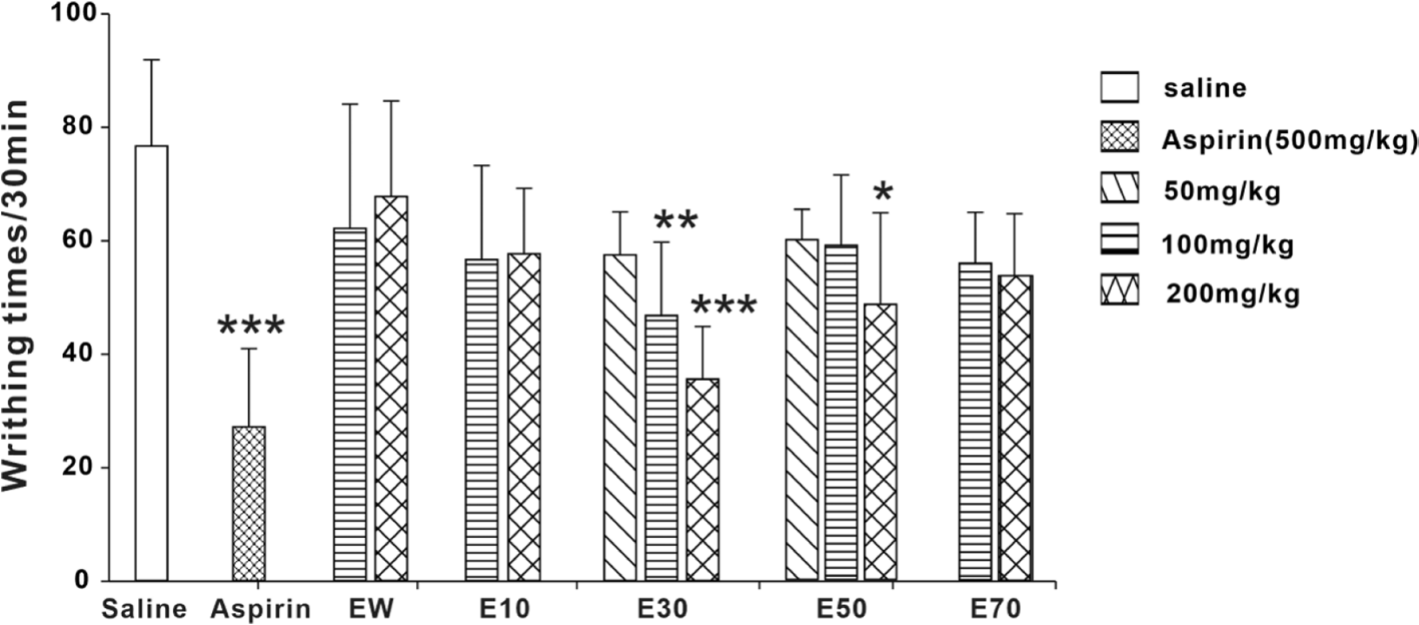
Effects of different eluted fractions from *P. scabra* on pain response induced by glacial acetic acid. EW: water elution fraction; E10: 10% ethanol elution fraction; E30: 30% ethanol elution site; E50: 50% ethanol elution fraction; E70: 70% ethanol elution fraction. *n* = 10 mice/group. The experimental data were expressed as “mean ± standard deviation”. ^***^p < 0.001, ^**^p < 0.01, ^*^p < 0.05 versus saline (kruskal-Wallis test)

As shown in Fig. 3, the positive control drug of Aspirin (500 mg/kg) could alleviate the painful responses induced by glacial acetic acid. E30 and E50 also reduced the writhing times of mice in a dose-dependent manner respectively. EW, E10 and E70 displayed no analgesic actions compared with saline.

### Effects of different elution fractions from *P. scabra* on formalin-induced pain

**Fig. 4:**
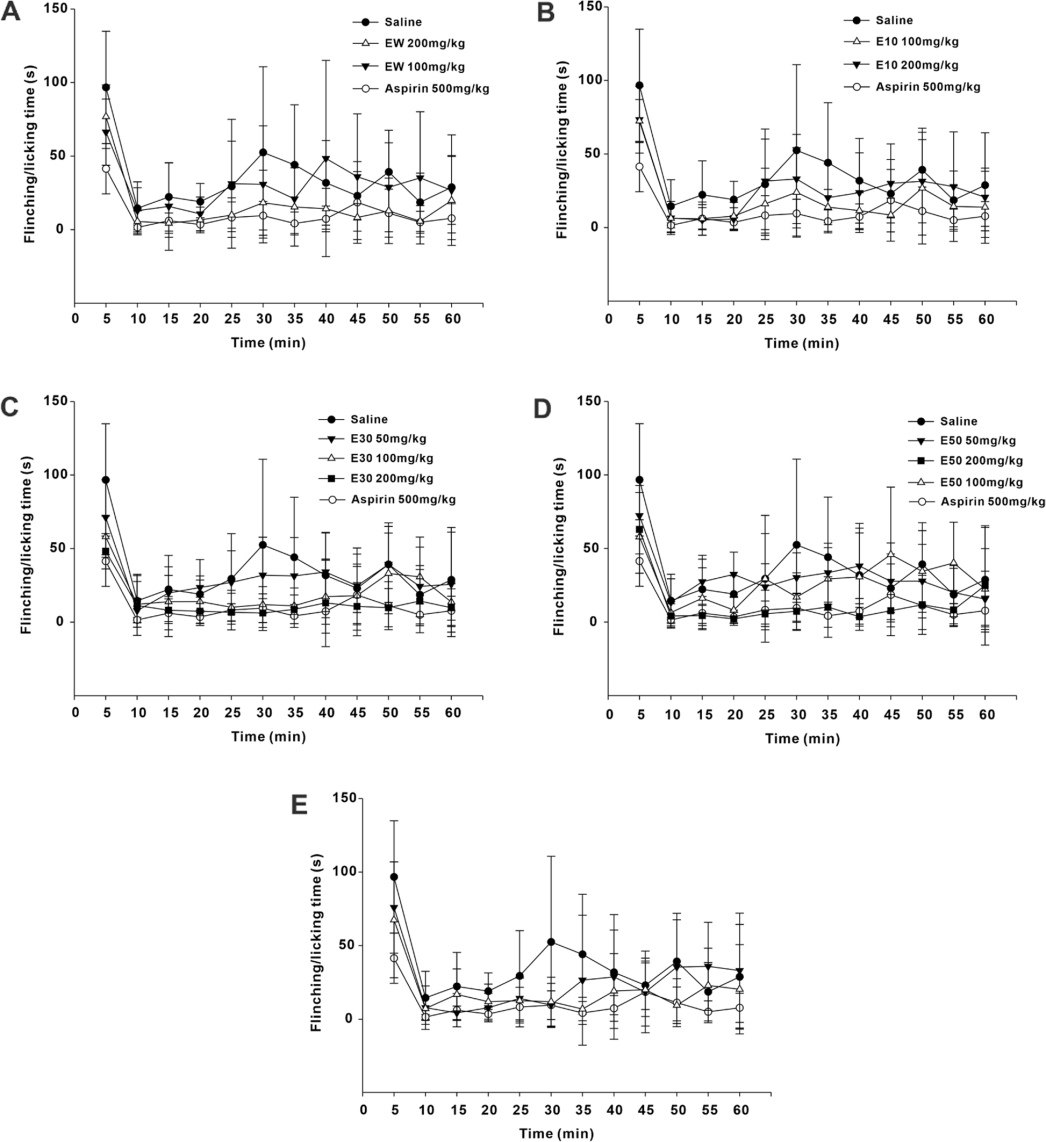
The change of pain response over time of mice in each group within 1 hour. Phase I (0 ~ 10 min), phase II (10 ~ 60 min). EW: water elution fraction; E10: 10% ethanol elution fraction; E30: 30% ethanol elution fraction; E50: 50% ethanol elution fraction; E70: 70% ethanol elution fraction. *n* = 10 mice/group. The experimental data were expressed as “mean ± standard deviation”

The pain induced by formalin can be divided into phase I (0 ~ 10 min) and phase II (10 ~ 60 min), phase I is caused by peripheral mechanism and phase II is by central mechanism. Figure 4 showed the change of pain response over time of mice in each group within 1 hour.

**Fig. 5:**
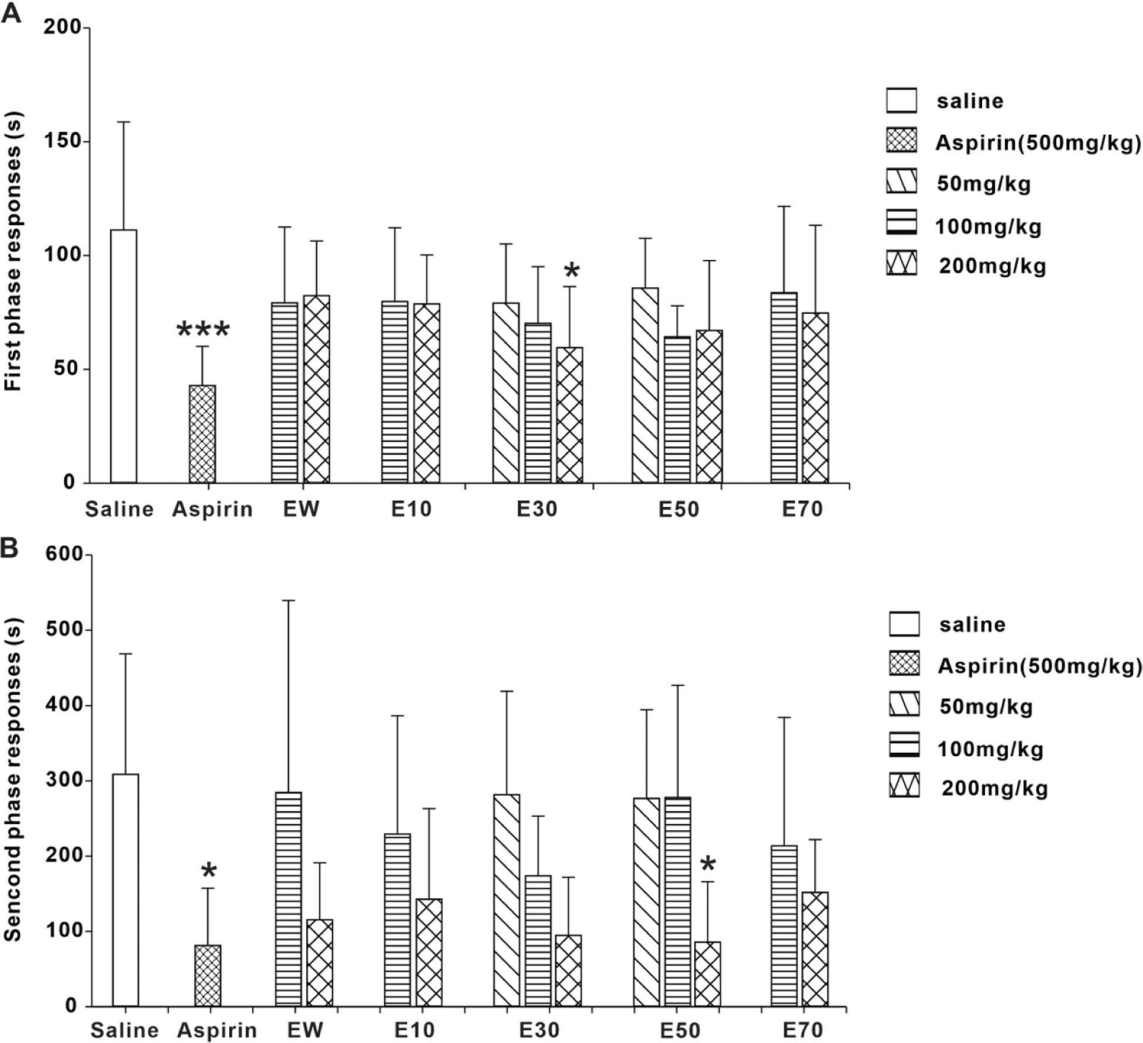
Effects of different elution fractions from *P. scabra* on pain response of phase I (Fig. 5A) and phase II (Fig. 5B) induced by formalin. EW: water elution fraction; E10: 10% ethanol elution fraction; E30: 30% ethanol elution fraction; E50: 50% ethanol elution fraction; E70: 70% ethanol elution fraction. *n* = 10 mice/group. The experimental data were expressed as “mean ± standard deviation”. ^***^p < 0.001, ^**^p < 0.01, ^*^p < 0.05 versus saline (kruskal-Wallis test)

As shown in Fig. 5A, both Aspirin (500 mg/kg) and E30 had the inhibitory effects on the paw flinching and licking time of formalin-injected mice during the phase I (0 ~ 10 min). E30 could alleviate the pain response of phase I in a dose-dependent manner. No analgesic activites were observed with EW, E10, E50 and E70 in phase I.

Figure 5B indicated that E50 could dose-dependently inhibite the formalin-induced paw flinching and licking time of mice during the phase II (10 ~ 60 min). Aspirin (500 mg/kg) also attenuated the pain response of phase II. EW, E10, E30 and E70 generated little effects on the painful responses in phase II.

### Effects of different elution fractions from *P. scabra* on the inflammatory response induced by carrageenan

**Fig. 6:**
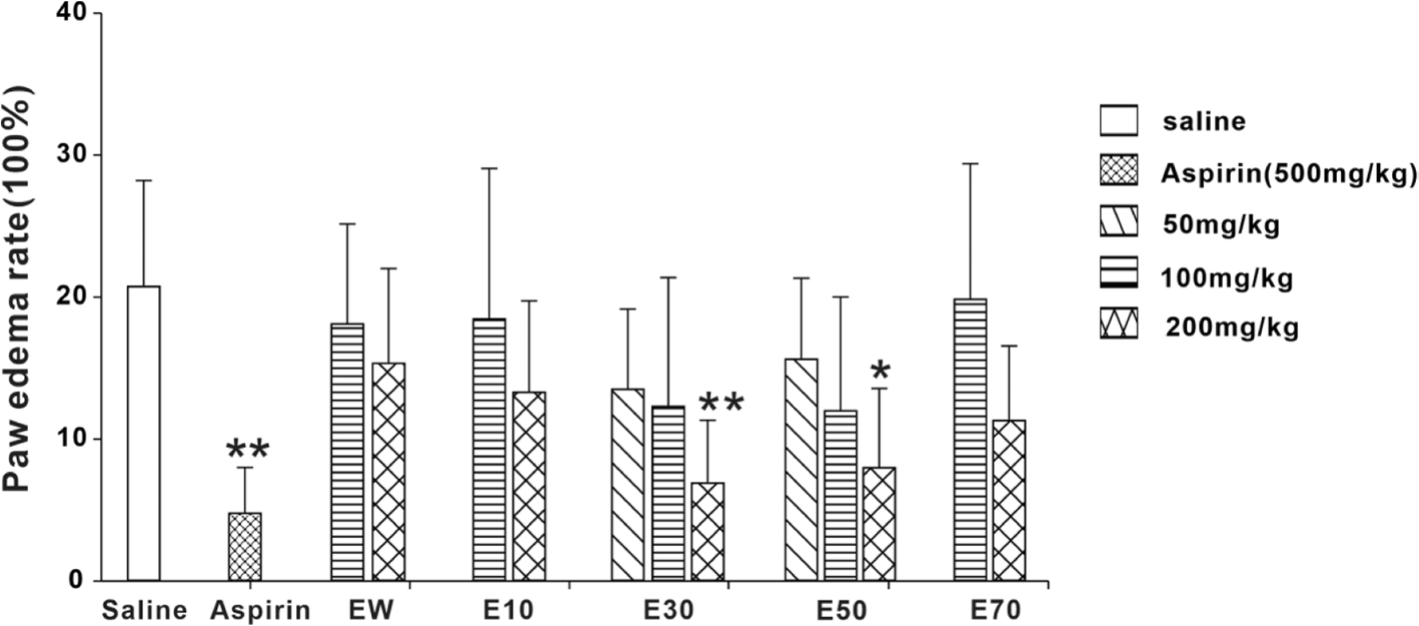
Effects of different eluted parts from *P. scabra* on carrageenan-induced paw edema rate in mice. EW: water elution fraction; E10: 10% ethanol elution fraction; E30: 30% ethanol elution fraction; E50: 50% ethanol elution fraction; E70: 70% ethanol elution fraction. *n* = 10 mice/group. The experimental data were expressed as “mean ± standard deviation”. ^***^p < 0.001, ^**^p < 0.01, ^*^p < 0.05 versus saline (kruskal-Wallis test)

**Fig. 7:**
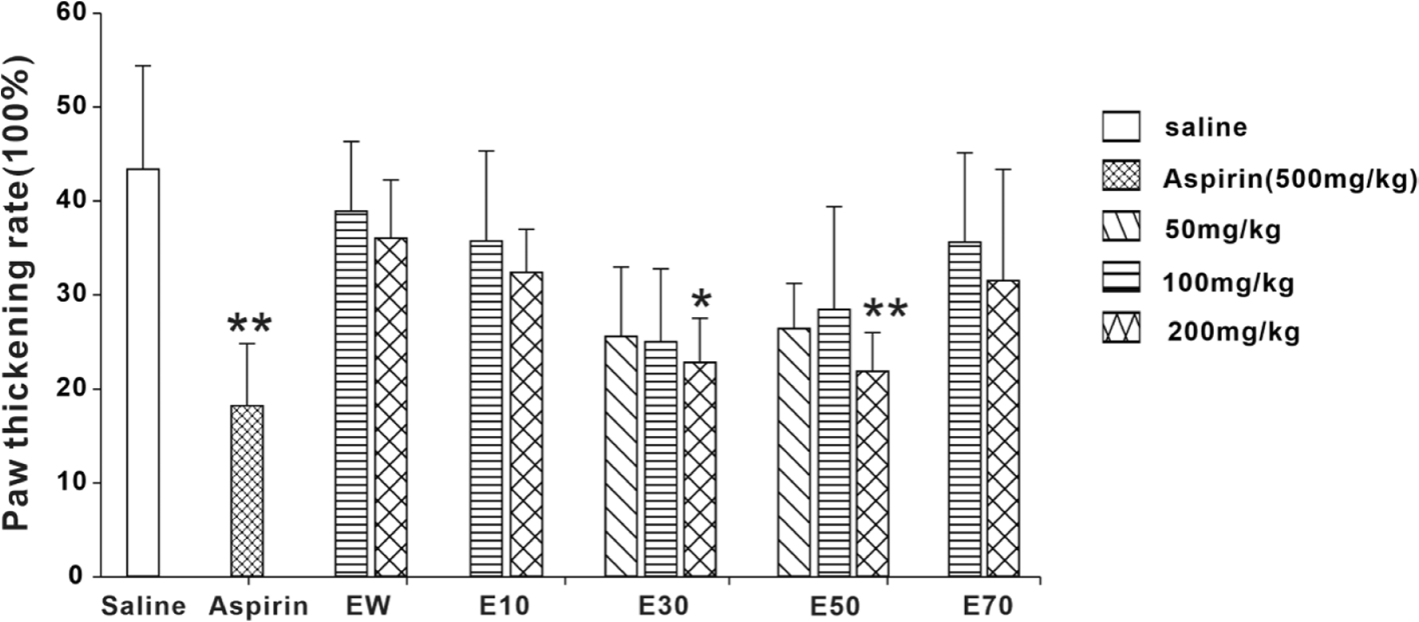
Effects of different eluted parts from *P. scabra* on carrageenan-induced paw thickening rate in mice. EW: water elution fraction; E10: 10% ethanol elution fraction; E30: 30% ethanol elution fraction; E50: 50% ethanol elution fraction; E70: 70% ethanol elution fraction. *n* = 10 mice/group. The experimental data were expressed as “mean ± standard deviation”. ^***^p < 0.001, ^**^p < 0.01, ^*^p < 0.05 versus saline (kruskal-Wallis test)

As shown from Fig. 6 and Fig. 7, Aspirin (500 mg/kg), E30 and E50 could effectively reduce the paw swelling and paw thickening rate induced by carrageenan in mice. The inhibitions produced by E30 and E50 were dose-dependent. EW, E10 and E70 showed no significant effects on carrageenan-induced inflammation.

### Effects of different elution fractions from *P. scabra* on the inflammatory response induced by xylene

**Fig. 8:**
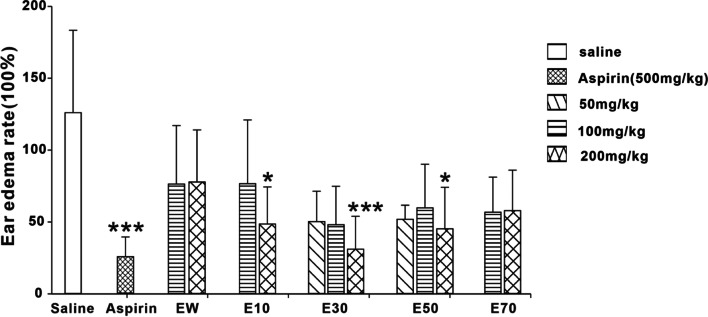
Effects of different eluted parts from *P. scabra* on xylene-induced ear edema rate in mice. EW: water elution fraction; E10: 10% ethanol elution fraction; E30: 30% ethanol elution fraction; E50: 50% ethanol elution fraction; E70: 70% ethanol elution fraction. *n* = 10 mice/group. The experimental data were expressed as “mean ± standard deviation”. ^***^p < 0.001, ^**^p < 0.01, ^*^p < 0.05 versus saline (kruskal-Wallis test)

**Fig. 9:**
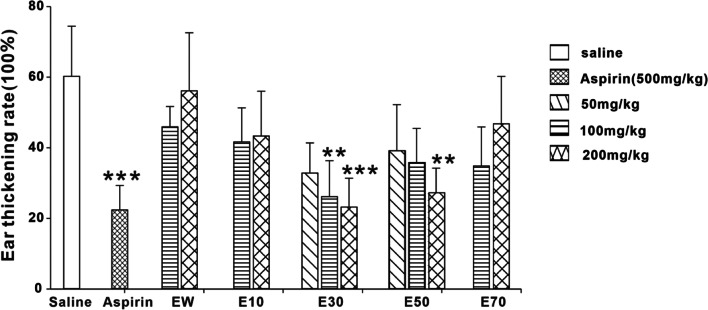
Effects of different eluted parts from *P. scabra* on xylene-induced ear thichening rate in mice. EW: water elution fraction; E10: 10% ethanol elution fraction; E30: 30% ethanol elution fraction; E50: 50% ethanol elution fraction; E70: 70% ethanol elution fraction. n = 10 mice/group. The experimental data were expressed as “mean ± standard deviation”. ^***^p < 0.001, ^**^p < 0.01, ^*^p < 0.05 versus saline (kruskal-Wallis test)

Compared with saline control, Aspirin (500 mg/kg), E30 and E50 all reduced the ear swelling and ear thickening rate caused by xylenes in mice (Fig. 8 – Fig. 9). The inhibitions produced by E30 and E50 were dose-dependent. E10 showed some certain activity. EW, and E70 showed no anti-inflammation effects.

## Discussion

In our previous studies, certain concentration of methanol and ethanol were taken as solvents to extract total iridoid glycosides from *P. scabra* through the ultrasonic and hot-refluxing methods, and the total iridoid glycosides were reached as 70 mg/g and 82 mg/g, respectively. Both of the two methods were time-consuming. Considering the cost and environmental pollution, the economical and environmentally friendly method of ultrasonic-microwave synergistic extraction (UMSE) was applied in this article. UMSE makes full use of high-energy effect of microwave and ultrasonic cavitation [27], as a consequence having the advantages in promotion of cell division and reinforcement of mass transfer. Thus, this method has been widely used for the adequate extraction of active substances from herbal medicine [28]. In addition, response surface methodology (RSM) is widely applied in the optimization of extraction processes [29], that was mainly devoted to develop and improve the design process ultimately by establishing a mathematical model under the reasonable assessment of hydrolysis parameters [30]. In this article, single-factor statistical method and RSM were combined together to optimize UMSE for *P. scabra*, so as to improve the content of total iridoid glycosides in *P. scabra* extract.

In the primary stage of pharmacological experiments, the dose concentration of each elution fraction were 100 mg/kg and 200 mg/kg respectively. At these two doses, E30 and E50 were found to have analgesic and anti-inflammatory activities, while EW, E10 and E70 had no effects. To further investigate the dose range, 50 mg/kg of E30 and E50 was also tried in the subsequent experiments. The writhing reaction of mice induced by glacial acetic acid belongs to chemical stimulation. Intraperitoneal injection of acetic acid promotes the release of visceral inflammatory mediators such as prostaglandins and cytokines (IL-8, TNF- α, and IL-1 β), which elicits writhing behaviors in mice [31]. The pain model of acetic acid is widely used to evaluate the analgesic activity of central and peripheral analgesics [32]. Our present study showed that the higher dose (200 mg/kg) of E30 and E50 could significantly reduce the writhing times of mice caused by acetic acid. It was suggested that E30 and E50 could generate analgesic effects from the periphery or center. Next, the formalin test was carried out to verify the above result. The painful responses induced by formalin consists of two phases with different pain mechanisms. The Phase I (acute neurogenic pain) occurs within seconds after intra-plantar injection of formalin. Mice exhibit paw-licking behavior due to chemical stimulation of the peripheral terminals of nociceptors. The painful behaviors during phase II (inflammatory pain) result from both peripheral and central sensitization [33–36]. Our data demonstrated that E30 and E50 could significantly reduce the paw flinching and licking time of mice in phase I and phase II, respectively. Therefore, it was reasonably predicted that E30 might act centrally to exert an analgesic action, while E50 might take effects peripherally.

Carrageenan-induced acute inflammation in the rat hind paw oedema model was first introduced by Winter et al. It has been widely used as a preliminary screening test for new anti-inflammatory drugs, mainly Non-Steroidal Anti-Inflammatory Drugs [37]. The experimental model exhibits a high degree of reproducibility [38]. The pro-inflammatory cytokines (NO, PGE2, TNF-α, IL-1β, IL-6) and free radicals will be released with the injection of carrageenan, accompanied by the acute inflammation with edema. Simultaneously, the carrageenan-induced inflammatory process also involves the oxidative stress and reactive oxygen species (ROS) production, along with the reduction of antioxidant enzyme activities (such as SOD, CAT, and GSH-Px), the generation of free radicals and lipid peroxidation [39, 40]. Xylene-induced ear swelling in mice is also a classic model for evaluating the anti-inflammatory activity of drugs. This acute model causes acute inflammation by inducing the release of inflammatory mediators (such as histamine, 5-hydroxytryptamine and bradykinin) and promoting capillary permeability, thus eventually leading to edema [41, 42]. Xylene-induced edema is also partly associated with the release of substance P and causes neurogenic inflammation, such as redness, warmth, swelling and hypersensitivity. In the course of neurogenic inflammation, substance P and calcitonin gene-related peptide (neuropeptides) evokes the release of histamine from the mast cells, in turn, histamine also induces the release of substance P and CGRP. Both of these mechanisms are inter-related with each other in causing neurogenic inflammation [43, 44]. It was clearly found that the E10 fraction could reduce the edema induced by xylene but not by carrageenan, the reason may be that there are some differences in the inflammatory mechanisms between them. In our study, both E30 and E50 could significantly reduce the carrageenan-induced paw edema and xylene-induced ear swelling in a dose-dependent manner, indicating that both of them had good anti-inflammatory effects.

The current researches on *P. scabra* were mainly focused on the chemical constituents and biological activities, there were few studies on the improvement of extraction process. As the main components of *P. scabra*, iridoid glycosides could acted as indicator to optimize the extraction process of *P. scabra* using UMSE. The previous research on biological activity was mainly concentrated in anti-tumor and immunity enhancement. In recent years, the anti-inflammatory effects of iridoids from *P. scabra* in vitro have aroused people’s more attention. The other herbal plants that belong to the same category of *P. scabra* have been studied on the analgesic and anti-inflammatory activities. For example, the iridoids of *Patrinia heterophylla* was reported to have the anti-inflammatory effects, which could inhibit NO activities and reduce the ROS production through *vitro* and *vivo* experiments [45]. *Patrinia villosa* was found to show strong anti-inflammatory and anti-nociceptive effects through a series of animal experiments including the ear edema test, carrageenan-induced paw edema test, cotton pellet-induced granuloma formation test, and acetic acid-induced writhing test. *Patrinia villosa* was also investigated to have a good effect on pelvic inflammation [15]. *Patrinia scabiosaefolia* had the therapeutic effect of ulcerative colitis in mice by effectively down-regulating the productions and expressions of inflammatory mediators [46]. Our study also provided a further basis for the analgesic and anti-inflammatory of *P. scabra*.

The above study belonged to the preliminary research of our project, in which the analgesic and anti-inflammatory effects of *P. scabra* were confirmed and active fractions were also screened out. Combined with the previous research, the article reasonably speculated that iridoid glycosides played a significantly important role in the pharmacodynamic effect of *P. scabra.* However, the composition of Traditional Chinese medicine is characterized by complexity and diversity, it was difficult to completely justify the biological effects of *P. scabra* only on the basis of iridoid glycosides. The role of other ingredients (such as flavonoids, lignans) should not be overlooked, there may be synergistic or antagonistic effects between them. In the future, our team plan to separate and purify the screened active fractions, to explore the biological activity of isolated monomer compounds, so as to clarify the substance basis of anti-inflammatory and analgesic.

## Conclusion

Taken together, the ultrasonic-microwave synergistic extraction was applicable to enhance the content of total iridoid glycosides from *P. scabra*. The optimized conditions were as follows: 52% of ethanol, 1:18 g/ml of material-to-liquid ratio, microwave power at 610 W and extraction time in 50 min. The content of PSI increased significantly after gradient elution by the macroporous resin. Pharmacological assays showed that 30 and 50% ethanol elution fractions of *P. scabra* exhibited good analgesic and anti-inflammatory activities. E30 and E50 might relieve pain centrally and peripherally, respectively. This current study provides some scientific evidence for the utilization of the extracts from *P. scabra* plant as an anti-inflammatory and analgesic agent. It is necessary to clarify the substance basis and mechanism in the future.

## Data Availability

The datasets generated and/or analyzed during the current study are available from the corresponding author on reasonable request.

## Abbreviations

UMSE: Ultrasonic-microwave synergistic extraction
RSM: Response surface methodology
PSI: *Patrinia scabra* total iridoid glycosides
EW: Water elution fraction of *patrinia scabra* extract
E10: 10% ethanol elution fraction of *patrinia scabra* extract
E30: 30% ethanol elution fraction of *patrinia scabra* extract
E50: 50% ethanol elution fraction of *patrinia scabra* extract
E70: 70% ethanol elution fraction of *patrinia scabra* extract
